# Psychological stress, wellbeing, and coping strategies among mainland Chinese students in Malaysia’s multilingual context: a qualitative study

**DOI:** 10.3389/fpsyg.2026.1843608

**Published:** 2026-06-04

**Authors:** Wenou Xue, Maodong Tian

**Affiliations:** 1 Qingdao Hengxing University of Science and Technology, Qingdao, China; 2 School of Literature, Panzhihua University, Panzhihua, China

**Keywords:** Chinese international students, coping strategies, English-medium instruction, multiculturalism, multilingualism, psychological stress, wellbeing

## Abstract

**Introduction:**

Malaysia’s English-medium universities operate within a multilingual and multicultural environment where English, Bahasa Melayu, and diverse cultural traditions intersect. For mainland Chinese postgraduates, adaptation involves not only academic demands but also shifting communicative expectations across academic, administrative, and sociocultural domains. While previous studies have focused mainly on academic and sociocultural adjustment, less attention has been devoted to psychological stress and coping within multilingual EMI environments.

**Methods:**

This qualitative study explored psychological stress, wellbeing, and coping strategies among mainland Chinese postgraduate students in Malaysia. Semi-structured interviews were conducted with 13 participants from four public and one private university. Interviews were conducted in Mandarin Chinese, transcribed verbatim, translated into English, and analyzed using thematic analysis with MAXQDA 2020 following Braun and Clarke’s six-phase framework.

**Results:**

Psychological stress emerged through the interaction of academic pressure, multilingual communication demands, and sociocultural exclusion. Delayed supervisory feedback, unclear expectations, diverse English accents, unfamiliar pedagogical practices, and Malay-mediated administration contributed to uncertainty and cognitive strain. Experiences of stereotyping, exclusion, and fragile belonging further intensified anxiety, insomnia, homesickness, and depressive feelings. Participants mainly relied on self-management, co-national peer support, family contact, religious beliefs, and resilience to cope with prolonged stress.

**Discussion:**

The findings indicate that psychological wellbeing in multilingual EMI environments is closely associated with institutional clarity, intelligibility, and accessibility across linguistic and cultural boundaries. The study conceptualizes stress as emerging within a multilingual EMI environment where communication, institutional access, supervision, and belonging operate as interconnected dimensions of adaptation.

## 1 Introduction

Malaysia has increasingly emerged as a major regional education hub, with universities offering a wide range of English-medium instruction (EMI) programs that have strengthened the country’s role as a key centre for EMI provision in Southeast Asia (Lo and Othman, 2023). This development has attracted a steadily growing number of international students. According to Education Malaysia Global Services (EMGS, 2024), the top five source countries of international students are China, Bangladesh, Indonesia, India, and Pakistan. Among them, Chinese students constitute the largest international cohort, with 33,216 applications recorded in 2024, representing a 24.7% increase compared to 2023. The expansion of China–Malaysia higher education cooperation, together with Malaysia’s reputation as an affordable, culturally accessible, and geographically proximate study destination, has further contributed to the rising mobility of mainland Chinese students in recent years (Koh and Yeoh, 2024).

A defining characteristic of Malaysia’s higher education landscape is its multilingual and multicultural environment. Bahasa Melayu serves as the national language, while English functions as a second language and the primary medium of instruction in many postgraduate programs (Huiling and Ismail, 2022). The coexistence of multiple languages, diverse cultural traditions, and the significant influence of Islam on social and educational practices creates a distinctive context for international students’ academic and everyday experiences (Ebrahimi and Yusoff, 2020; Singh and Jack, 2022). Although this diversity offers valuable opportunities for intercultural learning and engagement, it also presents substantial challenges, as international students must negotiate unfamiliar cultural norms, religious practices, and communication patterns while simultaneously adapting to the academic demands of EMI environments.

International students are frequently exposed to psychological stressors when navigating unfamiliar cultural and academic contexts. These include academic pressure (Lian and Wallace, 2018; Lin et al., 2019; Wang, 2022), homesickness and loneliness (Li, 2023; Newton et al., 2021; Zhao et al., 2023), as well as anxiety and depression (Nguyen et al., 2019; Xie et al., 2019). They may also confront discrimination and stereotyping, which contribute to their sense of alienation and vulnerability (Dovchin, 2020; McDonough et al., 2022). Language anxiety and inadequate English proficiency intensify these difficulties, resulting in a persistent sense of strain in academic and social domains (Dongqi et al., 2020; Hor and Jusoh, 2021). To manage these pressures, students employ a variety of coping strategies, ranging from constructive approaches such as exercise, listening to music, and relaxation (De Moissac et al., 2020; Cao et al., 2018) to less active responses such as endurance, avoidance, and withdrawal (Cao et al., 2022; Yu and Leung, 2023).

While earlier studies frequently grouped mainland Chinese students with those from Hong Kong and Taiwan, such categorization overlooks the distinct educational systems, linguistic environments, and cultural traditions that shape their academic trajectories (Li et al., 2018; Philips, 2021). Unlike their Hong Kong and Taiwanese counterparts, who are socialized in bilingual or multilingual settings that emphasize critical thinking and independent learning, students from mainland China enter higher education abroad with backgrounds shaped by exam-oriented instruction, Confucian values of authority and harmony, and limited opportunities for authentic English communication. These differences position mainland Chinese students as a unique group.

Nonetheless, in non-English-speaking or multilingual contexts such as Malaysia, research on the psychological stress and wellbeing of Chinese international students remains underexplored. Existing scholarship has primarily examined academic adjustment, English language proficiency, and sociocultural integration (Hor and Jusoh, 2021; Rathakrishnan et al., 2021; Zhai and Razali, 2022; Zhang and Wahab, 2022), yet relatively little attention has been devoted to the psychological dimensions of students’ experiences and the coping mechanisms they employ. This gap is particularly salient in Malaysia, where the coexistence of multiple languages, diverse cultural traditions, and religious practices creates distinctive pressures that differ from those encountered in Anglophone host countries.

Unlike Anglophone destinations where English functions as the relatively stable language of institutional communication, Malaysia’s multilingual EMI environment requires students to navigate shifting linguistic expectations across academic, administrative, and sociocultural domains. Students must simultaneously manage diverse English accents, Malay-mediated institutional procedures, multilingual peer interaction, and culturally differentiated expectations within EMI settings.

Rather than treating academic, linguistic, and sociocultural challenges, this study conceptualizes psychological stress as emerging within a multilingual EMI ecology in which communication, institutional access, and social participation are shaped across linguistic and cultural boundaries. In Malaysia, mainland Chinese students must navigate English-medium academic work, diverse English accents, Malay-mediated administrative procedures, and ethnically segmented peer interaction. Unlike many Anglophone destinations, where English functions as the relatively stable language of academic and institutional communication, Malaysia’s multilingual EMI environment requires students to negotiate shifting linguistic expectations across academic, administrative, and social domains. The study thus demonstrates that stress emerges through layered communication demands, rather than simply reproducing familiar stressors in a different setting. The following research questions guide this study:

*RQ1*. How do stressors encountered in Malaysia’s multilingual EMI context influence the psychological stress and wellbeing of mainland Chinese students?

*RQ2*. What coping strategies do mainland Chinese students adopt to manage stress and sustain their wellbeing in Malaysia’s multilingual EMI context?

## 2 Literature review

### 2.1 Psychological stress and communication in EMI contexts

Psychological stress among international students is increasingly understood to arise from the interaction among academic demands, sociocultural transition, and institutional communication processes rather than from isolated adjustment difficulties alone (Han et al., 2024; Newton et al., 2021; Tan and Greenwood, 2021). Previous studies have shown that distress intensifies when academic demands exceed perceived coping resources, particularly during periods of prolonged uncertainty and limited support (Maharaj et al., 2024; Maharaj et al., 2024; Zhu et al., 2021). However, much of this research has been conducted in Anglophone contexts where English functions as the dominant institutional language, leaving multilingual EMI environments comparatively underexplored.

Discrimination further shapes students’ psychological experiences within EMI contexts. Linguistic differences, particularly accent, frequently function as markers of exclusion and marginalization (Lan et al., 2023; McDonough et al., 2022). Research from Anglophone countries documents accent-based judgment, social exclusion, and xenophobic labelling that contribute to reduced self-confidence and psychological distress among international students (Dovchin, 2020; Lam and Hale, 2024; Yeo et al., 2019). In multilingual EMI environments, these tensions may become more complex as students navigate multiple English varieties, multilingual peer interaction, and locally mediated communication practices (Xue, 2026).

### 2.2 Coping and multilingual EMI contexts

Coping strategies are closely shaped by sociocultural context. Students from collectivist backgrounds often equate academic achievement with self-worth, which may intensify stress and discourage formal help-seeking (Ma et al., 2020). As a result, informal, digital, and peer-based support systems are frequently used to compensate for limited institutional support (Atherton and Cornwall, 2022; Park et al., 2025; Zhao, 2025). Recent qualitative research further suggests that stress and coping among Asian international students are deeply shaped by family expectations, emotional endurance, and future-oriented responsibility (Le and Trinh, 2025).

Existing research has paid comparatively limited attention to how multilingual environments reshape psychological adaptation in EMI higher education. In Malaysia’s multilingual EMI context, students must navigate shifting linguistic boundaries across English-medium academic communication, Bahasa Melayu-mediated administrative practices, and ethnically differentiated social interaction (Huiling and Ismail, 2022; Tuerxun et al., 2020; Zhai and Razali, 2022; Zhang and Wahab, 2022; Zhou and Pazil, 2025). These layered communication demands may fundamentally reshape how stress, belonging, and coping are experienced within higher education settings.

Previous studies conducted in Malaysia have identified challenges related to language barriers, academic adjustment, and limited social integration among international students (Rathakrishnan et al., 2021). However, much of this scholarship remains primarily descriptive, offering limited analytical attention to the mechanisms by which the demands of multilingual communication shape psychological wellbeing in EMI higher education. In addition, research specifically focusing on mainland Chinese students remains methodologically fragmented and insufficiently grounded in in-depth, student-centred qualitative inquiry (Zhao et al., 2023; Shanshan et al., 2023).

Existing scholarship has also paid limited attention to how psychological adaptation is negotiated within multilingual EMI environments where academic participation, institutional communication, and interpersonal belonging are mediated across multiple linguistic and cultural boundaries. While previous research has examined academic and sociocultural adaptation among international students in Malaysia, less attention has been devoted to how multilingual communication demands intersect with supervision, belonging, and coping processes to shape psychological wellbeing. More specifically, there is limited understanding of how mainland Chinese postgraduate students experience, interpret, and negotiate psychological stress within Malaysia’s multilingual EMI context, where access to participation, institutional support, and social integration are unevenly shaped by overlapping linguistic, cultural, and institutional conditions.

## 3 Theoretical framework

This study draws on Stress and Coping Theory (Lazarus and Folkman, 1984) and Self-Efficacy Theory (Bandura, 1997) to examine how international students experience and manage psychological stress in English-medium instruction contexts.

Stress and Coping Theory conceptualizes stress as arising from the appraisal of environmental demands relative to available resources. Primary appraisal concerns whether situations such as academic workload or language barriers are perceived as threats or challenges, while secondary appraisal involves the evaluation of coping resources, including language competence and social support. These appraisals shape coping responses, typically categorized as problem-focused or emotion-focused, which in turn influence psychological outcomes (Teh et al., 2023; Rathakrishnan et al., 2022).

Self-Efficacy Theory complements this framework by emphasizing individuals’ beliefs in their capacity to manage challenges. Higher self-efficacy is associated with more adaptive appraisals and greater use of proactive coping strategies, whereas lower self-efficacy is linked to avoidance and increased vulnerability (Kristensen et al., 2023; Sabouripour et al., 2021; Rosli and Saleh, 2023).

Importantly, self-efficacy informs both primary and secondary appraisal processes, thereby shaping coping responses. Together, these frameworks provide an integrated analytical lens for examining how multilingual academic and sociocultural demands are appraised, how coping resources are perceived and mobilized, and how variations in self-efficacy shape students’ coping responses and psychological wellbeing within Malaysia’s multilingual EMI environment.

## 4 Methodology

### 4.1 Research design and sample

This study adopted a qualitative design grounded in the interpretivist paradigm, emphasizing individuals’ subjective meanings and lived experiences (O’Donoghue, 2018; Pervin and Mokhtar, 2022). This approach is well-suited to examining how participants interpret and respond to psychological stress within specific sociocultural and educational contexts.

Participants were recruited using purposive sampling, targeting mainland Chinese postgraduate students with direct experience in English-medium instruction (EMI) programs. To enhance diversity, maximum variation sampling was applied across academic disciplines, institutional types, and demographic backgrounds (Barbour, 2001; Sandelowski, 1995).

The final sample comprised 13 participants from five Malaysian universities (four public and one private), aged 20–40, representing multiple regions of China. Participation was voluntary, and pseudonyms were used to ensure confidentiality. Sample size was determined based on data saturation, defined as the point at which no new codes or themes emerged.

Table 1 presents an overview of participants’ profiles, illustrating the diversity of academic backgrounds, institutional affiliations, and demographic characteristics.

**Table 1:** Participants’ profiles.

Pseudonym	Gender	Degree level	Age range	Institution type	Field of study	Length of stay in Malaysia
Abby	Female	PhD	35–40	Private	Education	4 years
Alex	Male	PhD	35–40	Public	Management	5 years
Bianca	Female	Master	25–30	Private	Applied Linguistics	1 year
Bruce	Male	Master	25–30	Public	Engineering	1 year
Chen	Female	PhD	35–40	Public	Education	4 years
Ken	Female	PhD	30–35	Public	Social Sciences	3 years
Leo	Male	PhD	30–35	Public	Computer Science	4 years
Mimi	Female	Master	20–25	Public	Business	1 year
Ped	Male	PhD	35–40	Public	Management	5 years
Sean	Male	Master	20–25	Private	Media Studies	1 year
Serena	Female	PhD	30–35	Public	Education	6 years
Wang	Female	PhD	25–30	Public	Applied Linguistics	3 years
Yassen	Male	Master	20–25	Public	Engineering	2 years

### 4.2 Instrument

Data were collected through semi-structured interviews with open-ended questions, enabling participants to articulate their experiences, perceptions, and coping processes in depth (Kvale, 1996; Ruslin et al., 2022).

The interview protocol was developed based on the literature on psychological stress, wellbeing, and coping. Questions focused on stress experiences, emotional responses, perceived stressors, and coping strategies in EMI contexts. Probing questions were used to elicit further clarification and depth (Mann, 2011).

To enhance content validity, the protocol was reviewed by experts in international education and qualitative research. A pilot interview was conducted to refine question clarity and sequencing prior to data collection.

### 4.3 Procedure and ethical considerations

Participants were recruited through a voluntary response sampling strategy. An invitation was posted on Xiaohongshu (Rednotes), a Chinese social media platform widely used by mainland Chinese students to share overseas study experiences. Interested individuals contacted the researcher directly, ensuring that participation was voluntary and self-initiated. However, this recruitment approach may have introduced self-selection bias, as students with stronger experiences of stress or adaptation challenges may have been more willing to participate.

Data collection was conducted between August and September 2025. As this period coincided with the summer vacation, all participants were temporarily residing in China at the time of the interviews. Consequently, interviews were conducted online via Tencent Meeting for reasons of accessibility and feasibility. Participants’ accounts were therefore based on retrospective reflections on their experiences in Malaysia rather than on immediate observation within the host context. At the same time, this temporal distance may have enabled participants to reflect more critically on their academic and sociocultural experiences after leaving the immediate institutional environment. Additionally, conducting interviews online allowed for greater flexibility in scheduling and participation, ensuring that students across different regions could take part. This approach also facilitated the inclusion of participants from diverse institutional backgrounds and study programs, thereby enhancing the richness of the data.

Before data collection, written informed consent was obtained from all 13 participants. The consent process included permission for audio recording and the anonymized use of data for research and publication purposes. Participants were fully informed of the study’s purpose, procedures, confidentiality measures, and their right to withdraw at any time without penalty.

According to the institutional ethics guidelines of the researcher’s affiliated university, formal ethics committee review was not required because the study involved voluntary adult participants and non-sensitive interview topics. An official exemption letter was issued by the institution. Written informed consent was obtained from all participants before data collection, and ethical standards regarding confidentiality, anonymity, and secure data management were maintained throughout the study. To protect participants’ identities, pseudonyms were used, and potentially identifiable information, such as exact age and province, was removed or generalized during transcription and reporting.

Interviews were conducted in Mandarin Chinese to facilitate natural expression (Mann, 2016). Each interview lasted between 20 and 45 min and was audio-recorded with participants’ consent. All interviews were transcribed verbatim, and notes were taken to capture contextual nuances.

To enhance the trustworthiness of the data, two professional translators independently translated the interview transcripts into English, and the translated versions were returned to participants for verification. This process helped ensure the linguistic accuracy and credibility of the interpretations.

### 4.4 Data analysis and trustworthiness

The analytic process began with repeated reading of transcripts and field notes to achieve familiarity with the data. Initial codes were generated line-by-line in MAXQDA 2020, capturing meaningful units related to psychological stress, wellbeing, and coping. For example, participant statements such as “my supervisor doesn’t reply, and I lose direction” were coded as “delayed supervisory feedback” and “loss of academic direction,” later grouped under the category “unclear supervisory communication” and integrated into the theme “faculty interaction.” Similarly, statements about difficulty understanding lecturers’ accents were coded as “accent comprehension difficulty” and included in the theme “language and pedagogy barriers.”

To enhance analytic consistency, two researchers independently coded a subset of transcripts, and discrepancies were resolved through discussion to refine the coding framework. Codes were then compared across all transcripts and organized into broader categories and candidate themes based on conceptual similarity. Themes were iteratively reviewed against both coded excerpts and the full dataset to ensure coherence, conceptual distinction, and alignment with the research questions.

Data saturation was monitored throughout; no new codes emerged after the tenth interview, confirming the sample was sufficient for thematic analysis (Braun and Clarke, 2006). Interviews were conducted in Chinese, and two professional translators independently translated the transcripts into English to ensure accuracy and maintain the integrity of participants’ meanings.

Table 2 presents illustrative examples of thematic coding, showing how raw interview excerpts were transformed into initial codes and overarching themes. This table is provided for demonstration purposes and does not represent the full dataset.

**Table 2:** Examples of thematic coding development.

Raw data excerpt	Initial code	Theme
“Because of the accent issues and also because professors here are very busy, if you message them on WhatsApp or email, they often do not reply.”	Delayed supervisory feedback	Faculty interaction
“I’ve noticed most people’s progress is quite slow -- slower than expected or what would be considered normal.”	Slow academic progress	Progress and workload pressures
“The professor had a very heavy Indian accent and spoke quickly. We were completely lost.”	Accent comprehension difficulty	Language and pedagogy barriers
“Even when linguistic comprehension was achieved, new modes of learning demanded constant adaptation.”	Pedagogical unfamiliarity	Language and pedagogy barriers
“Chinese students here cannot integrate. There is no friendly environment.”	Social exclusion	Sociocultural discrimination and belonging
“I feel no belonging here. I just want to finish my studies and return home.”	Fragile sense of belonging	Sociocultural discrimination and belonging
“It caused insomnia, hair loss - I could not sleep, especially during that initial period with the Research Methods course.”	Sleep disturbance	Psychological consequences of stress
“I try to plan my schedule and practice self-reflection to cope with stress.”	Self-regulation through planning	Self-management
“Mutual support with fellow doctoral students facing similar challenges proves valuable.”	Peer support	Peer and family support
“Buddhism provides me some solace. Its teachings… help me regulate my emotions.”	Religious coping	Religion and philosophy
“After much pressure, I learned when to relax, then make plans to achieve goals.”	Self-regulation and resilience	Resilience and growth

## 5 Findings

In response to the first research question, five themes emerged: faculty interaction, progress and workload pressures, language and pedagogy barriers, sociocultural discrimination and belonging, and psychological consequences of stress. For the second research question, four themes were identified: self-management, peer and family support, religion and philosophy, and resilience and growth.

**Figure 1:**
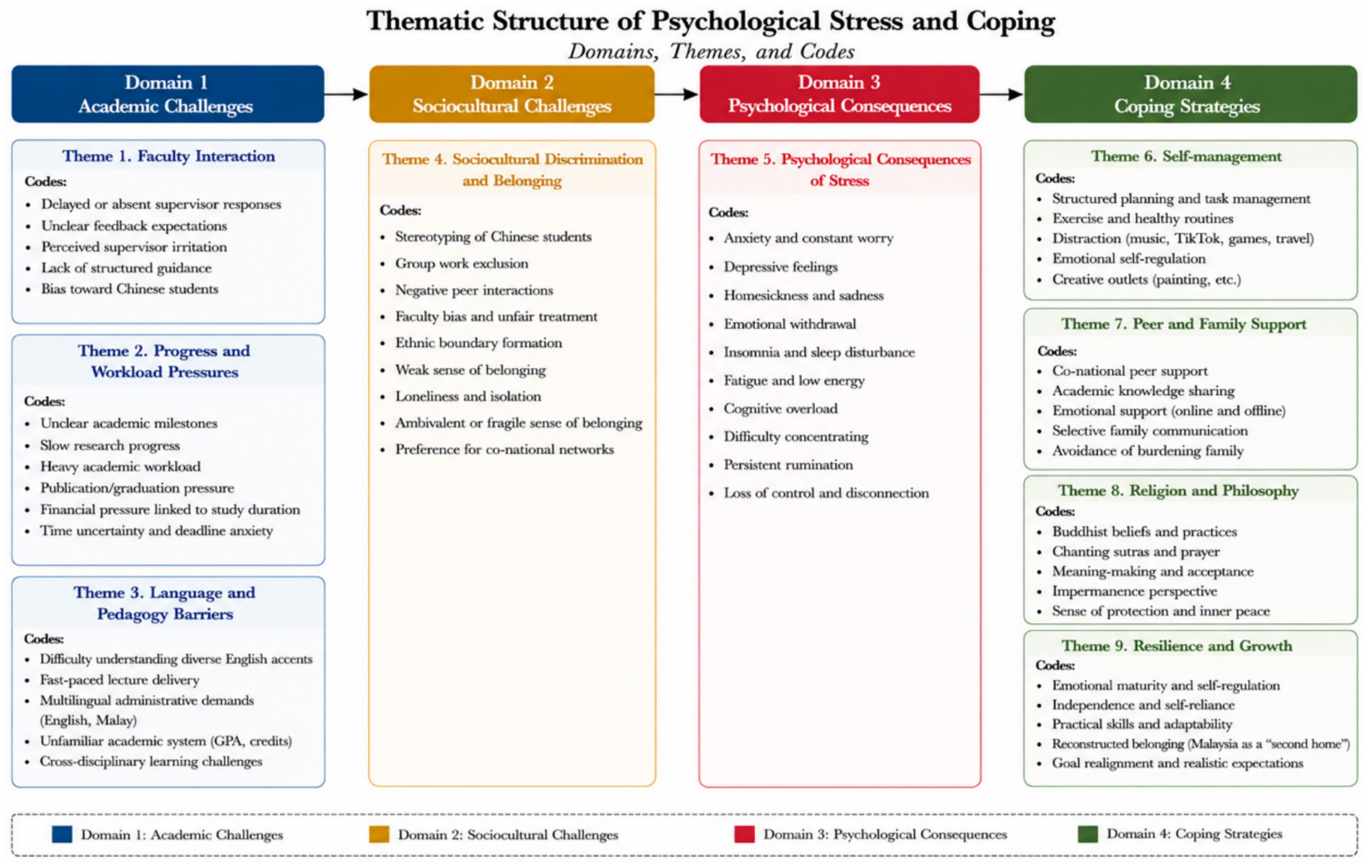
Thematic structure of psychological stress.

As illustrated in Figure 1, academic, linguistic, and sociocultural stressors interact within the multilingual EMI ecology to shape students’ psychological experiences, including anxiety, self-doubt, and cognitive overload, while also influencing their coping responses. The model conceptualizes stress as an interconnected EMI ecology rather than a series of isolated challenges.

### 5.1 Faculty interaction

Supervisory stress extended beyond delayed responses, reflecting a broader problem of relational opacity within Malaysia’s multilingual EMI ecology. Progress cues and evaluative standards were often communicated through varied accents and tacit mentoring norms, leading students to interpret open-ended feedback as indifference rather than autonomy. As Sean remarked, “Because of the accent issues and also because professors here are very busy, if you message them on WhatsApp or email, they often do not reply.” For Abby, this ambiguity translated directly into anxiety:

“*When the process flows smoothly, the pressure is manageable, and there is positive stress, as mentioned. But when my supervisor doesn’t reply, and I lose direction, that’s when anxiety arises.*”

Unclear expectations also shaped how feedback was experienced. Alex noted, “Most supervisors seem annoyed by students pushing them,” while iterative and inconsistent revisions intensified uncertainty:

“*When I submit a draft, they demand endless revisions. After multiple changes, they might say the first version was better. This back and forth creates tremendous pressure on Chinese students, affecting our mental state.*”

Perceived dismissiveness and bias further reduced trust. Mimi described how “delayed faculty feedback makes me wake up anxious daily”. Ped added, “My supervisor holds certain biases toward many Chinese students.”

“*In Malaysia, it is we who must make appointments with the lecturers, and they may not even be willing to meet us. It brings quite a lot of pressure.*”

These experiences indicate that supervisory stress is not merely workload related. These experiences reduced clarity during primary appraisal while weakening students’ perceived control and access to coping resources during secondary appraisal. Over time, diminished supervisory responsiveness undermined academic self-efficacy and intensified anxious monitoring within the multilingual EMI context.

### 5.2 Progress and workload pressures

Perceived slow progress often reflected opaque institutional signalling rather than individual delay. Milestones for proposals, publications, and graduation frequently became apparent only after mistakes, while administrative timelines intersected with visa and financial pressures. Under such conditions, time itself felt uncertain.

“*I’ve noticed most people’s progress is quite slow -- slower than expected or what would be considered normal. For instance, some students haven’t even completed their proposal defence after four years.*”

The burden extended beyond institutional demands to deeply personal pressures. Bianca observed, “Academic pressures, like thesis writing, are my main stressors.” Ken recalled, “During my first year, I felt stable, but later I started feeling time was pressing. Even my supervisor’s pressure was greater than external environmental pressures.” These accounts highlight how unpredictable timelines and supervisory expectations intensified feelings of falling behind.

As Chen explained, the intersection of financial and academic pressures was particularly acute.

“*I am under immense pressure. Studying in Malaysia involves financial stress and pressure to graduate. My daily interactions with other students are tense, and I feel constantly anxious and lost. Academically, I struggle to meet deadlines. I am anxious about graduating, and the pressures from various aspects of life have left me in a very poor state*.”

These conditions intensified students’ primary appraisal of academic threat while reducing perceived control over progress, graduation timelines, and financial stability. As confidence in managing competing academic and financial demands weakened, stress increasingly shifted from episodic pressure to chronic uncertainty.

### 5.3 Language and pedagogy barriers

Students’ challenges were less about general English proficiency and more about achieving intelligibility amid varied accents, rapid speech, and unfamiliar pedagogical methods. Accent variability and fast delivery increased cognitive load precisely when students were engaging in student-led critiques, cross-disciplinary tasks, and local assessment formats. Administrative interactions often required simultaneous competence in English and Malay, further intensifying processing demands and complicating daily study.

Sean illustrated this problem vividly: “The professor had a very heavy Indian accent and spoke quickly. We were completely lost.” Bianca recalled a similar experience: “Those of us who entered our university in 2023 encountered professors with strong accents, and the entire Chinese cohort felt tremendous pressure.” For some, accent difficulty was compounded by faculty perceptions. Bianca reflected, “Professors often questioned if we were truly graduate students, but our capabilities were fine.”

Pedagogical unfamiliarity added another layer of strain. Abby explained, “The challenge is the subject matter’s inherent difficulty, since I am doing cross-disciplinary research.” Even when linguistic comprehension was achieved, adapting to new learning modes required ongoing effort. Yassen shared how delivery mode shaped outcomes: “My GPA was particularly low in the first semester because it was online classes.” Serena stressed structural opacity, recalling:

“*The most frequent or significant pressure was academic. When I first arrived, I did not understand things like GPA, credits, or course planning. No one explained these to us, and there were no platforms like Xiaohongshu (Rednotes) where people shared their experiences or advice.*”

Daily learning was effortful, as students navigated varied English accents, fast speech, and Malay-mediated administrative procedures. Within this multilingual EMI ecology, comprehension itself became a persistent stressor rather than a neutral conduit to academic participation. The heightened cognitive load weakened communicative self-efficacy, reduced confidence in classroom interaction and supervisory communication, and contributed to increased uncertainty surrounding academic participation.

### 5.4 Sociocultural discrimination and belonging

Although Malaysia promotes itself as a multicultural society, participants often experienced diversity as mere co-presence rather than genuine intercultural engagement. Linguistic comfort zones aligned with ethnic boundaries, and unstructured group work frequently reinforced these divisions. Students reported stereotypes, social exclusion, and inequitable treatment on campus and in the wider community, creating psychological burdens that undermined academic confidence and social adjustment. Bruce recalled that one group member, a Kurdish student from Iraq, made a discriminatory remark during a group project, “Chinese people are lazy and lack creativity. Chinese people only deserve to use AI.”

Chen noted a similar experience: “Chinese students here cannot integrate. There is no friendly environment.” Others reported ambivalence or erosion of belonging. Ken reflected, “I am not someone with a strong sense of belonging, neither to Malaysia nor China.” Leo added, “It has eroded my sense of belonging. The initial result was self-doubt and passivity.”However, not all experiences were negative. With time, some students developed meaningful ties that reshaped their perceptions. Serena shared, “After many years here, I made friends and relationships. Now I feel Malaysia is like a second home.” Yassen expressed a similar view: “My sense of belonging in Penang remains strong. The city’s kindness outweighs the negativity.” Conversely, for others, the outcome remained disengagement. Mimi noted, “I feel no belonging here. I just want to finish my studies and return home.”

“*Some faculty openly tell local students that they dislike Chinese students because they generally think Chinese students’ English is poor and communication is difficult. This is their stereotype…The local stereotype of Chinese students is: poor English, clustering in Chinese-only groups.*”

Even interactions with Malaysian Chinese peers sometimes reinforced divisions through price discrimination or displays of superiority. These experiences produced a fragile sense of belonging, with some students maintaining a stronger attachment to China, while others remained ambivalent toward Malaysia. Experiences of exclusion and fragile belonging limited access to supportive social networks, increased reliance on co-national peers, and contributed to gradual social withdrawal within the multilingual EMI environment.

### 5.5 Psychological consequences of stress

Stressors across academic, linguistic, and sociocultural domains converged in persistent psychological and physical symptoms. Bruce described emotional distress: “I missed home a lot. During that time, I often cried for no reason.” He went further to explain, “It makes me feel unhappy and eager to leave. I want to return to China, to my family and friends.” For others, the effects were more physical. Yassen reflected, “Being forced to stay in Penang made me feel disconnected from my family for a long time.” The sense of disconnection produced feelings of isolation and loss of control over their situation.

Sleep disturbance was a recurring theme in students’ accounts:

“*It caused insomnia, hair loss - I couldn’t sleep, especially during that initial period with the Research Methods course. For one or two months, I was anxious every day, waking up thinking “Oh no, I still don’t understand this” or “How will I finish this assignment?” These thoughts constantly occupied my mind, creating anxiety that manifested physically - I couldn’t sleep, would wake up too early, and feel listless all day.*”

Leo noted, “The most notable effect has been sleeping disturbances, leading to diminished sleep quality.” Serena added, “The main issue was insomnia, staying up late worrying about these things.” Students associated these sleep problems with cycles of worry over academic performance, supervision delays, and uncertain timelines. The absence of restorative sleep further reinforced anxiety and fatigue, producing a feedback loop of declining wellbeing.

*“I’ve become more withdrawn and less social. It’s led to some depressive feelings. I think I’ve experienced mild depression, as have many of my peers. We often discuss how our progress is slower than expected.”*

The symptoms indicate movement from short-term strain to concrete risks for wellbeing, with clear costs for persistence and daily life. Rather than functioning as manageable episodes of stress, challenges accumulated into enduring psychological strain that shaped their sense of belonging, academic confidence, and overall wellbeing.

### 5.6 Coping through self-management

In response to cumulative academic and sociocultural pressures, students primarily relied on individualized coping strategies, including structured planning, task execution, routines, and distraction. These strategies helped maintain daily functioning but did not address underlying structural stressors. Bruce illustrated this pragmatic approach: “I either tough it out, distract myself by scrolling through TikTok, play games, or seek comfort in religion,” indicating reliance on short-term emotional relief. In contrast, Ken described a more problem-focused approach:

“*Regular exercise helps significantly, like morning runs, which release stress hormones. I also enjoy painting. It helps process events and calm my mind. Most importantly, identifying stress sources. For academic stress, making plans and completing tasks fundamentally resolves the anxiety.*”

Some students combined self-management with creative or social outlets. Serena shared, “I usually confided in close friends, which helped me feel better. Sometimes I listened to music or travelled.” For Yassen, food-related activities provided temporary distraction and comfort: “I distract myself with meals or cooking.” Despite their diversity, these strategies shared a common goal: reducing anxiety through personally controlled, manageable actions.

Self-management reflected a pragmatic orientation, emphasizing internal regulation over institutional support, which was often seen as linguistically demanding or culturally unfamiliar. While effective in the short term, this reliance on individual strategies also risked shifting responsibility from institutions to students, leaving structural barriers unchallenged. Stronger perceived self-efficacy was generally associated with greater engagement in planning, exercise, and task management, whereas lower confidence was more often linked to distraction and avoidance.

### 5.7 Peer and family support

Peer networks were the most accessible and reliable sources of support, offering both instrumental and emotional assistance when institutional support was limited. Co-national peers, particularly those at similar or more advanced stages, facilitated academic understanding and reduced uncertainty. Ken explained, “Peers, especially those ahead or at similar stages, discussing academics helps me identify gaps.” Similarly, Leo emphasized the collective dimension of peer solidarity: “Mutual support with fellow doctoral students facing similar challenges proves valuable.”

Emotional support also occurred through informal and digital interactions. Bruce highlighted virtual connections: “My friends provide emotional support mostly through online interactions,” which helped sustain morale and reduced feelings of isolation, even if interactions rarely extended beyond co-national circles.

Family support was intentionally limited due to geographic distance and to avoid worrying relatives in China. Serena explained,

“*I usually confided in close friends, which helped me feel better. I rarely shared with family, as I did not want them to worry. Sometimes, I would listen to music or travel with friends during breaks.*”

Peer and family support functioned as socially mediated coping resources that reduced uncertainty, strengthened emotional reassurance, and compensated for limited institutional support within the multilingual EMI environment.

### 5.8 Religion and philosophy

Religious and philosophical practices provided culturally grounded, emotion-focused coping strategies, helping students manage prolonged uncertainty. Rather than simply withdrawing from stress, these practices reframed challenges as meaningful and temporary, stabilizing emotional responses.

Bruce described how Buddhist beliefs supported this process:

“*Buddhism provides me with some solace. Its teachings, like recognizing the impermanence of suffering, help me regulate my emotions. Practices like chanting sutras or praying to Buddha make me feel accepted and protected, giving me a sense of security.*”

He added, “Practices like chanting sutras or praying to Buddha make me feel accepted and protected,” illustrating how routine practices created psychological security.

Beyond individual relief, religion and philosophy represented culturally situated strategies that reinterpreted stress within a broader moral and existential framework. These approaches demonstrate that coping is not purely cognitive but embedded in cultural meaning systems. These practices provided participants with broader interpretive frameworks through which stress could be understood and emotionally managed during periods of prolonged uncertainty.

### 5.9 Resilience and growth

Despite ongoing academic, sociocultural, and environmental stressors, many participants reported growth in independence, emotional maturity, and practical skills, often emerging after prolonged uncertainty and uneven support. Several described developing self-regulatory capacities, learning when to pause and when to act. Ken noted, “After much pressure, I learned when to relax, then make plans to achieve goals.”

Relationships and place-based experiences also reshaped students’ sense of belonging. Serena recounted, “My relationships and experiences here made me love this place… Malaysia became like a second home.” Similarly, Yassen noted:

“*Studying abroad taught me many skills, like plumbing and electrical work, since I prefer doing things myself. My resilience improved tremendously through handling challenges independently.*”

Resilience was expressed both through internal regulation and proactive action. Bianca remarked, “Discriminatory incidents fade since I won’t reencounter those people,” while Yassen stated, “I immediately reported them to our professors… suggested we maintain academic integrity.” These accounts suggest that resilience involves both internal regulation and proactive responses.

However, resilience was not effortless. Bruce proposed, “My advice is to prioritize your wellbeing… come here, get the degree, and leave. Don’t expect to stay long.” Ken similarly suggested, “Protect yourself… If Malaysia is necessary, learn local languages.”

These accounts suggest that resilience is context-dependent and shaped by structural conditions. Gains in self-reliance and adaptive meaning-making often emerged alongside prolonged stress and limited institutional support, indicating that resilience frequently developed out of necessity rather than institutional enablement. Resilience emerged less as an individual trait than as an adaptive response to prolonged institutional ambiguity, multilingual demands, and uneven access to support.

## 6 Discussion

This study conceptualizes psychological stress among mainland Chinese postgraduates in Malaysia as emerging within a multilingual EMI environment rather than from isolated academic, linguistic, or sociocultural variables alone. Students encounter EMI classrooms, diverse English accents, Malay-mediated administrative systems, and culturally differentiated social norms (Ebrahimi and Yusoff, 2020; Lo and Othman, 2023; Singh and Jack, 2022). Unlike many Anglophone destinations, Malaysia’s multilingual EMI environment requires students to navigate shifting communicative and cultural expectations across academic, administrative, and social domains (Huiling and Ismail, 2022). This layered environment reshapes how demands and available resources are appraised. When comprehension, supervision, and institutional access are negotiated across multiple linguistic and cultural contexts, perceived threat may increase while perceived control may diminish (Lazarus and Folkman, 1984), contributing to anxiety, sleep disturbance, and withdrawal (Han et al., 2024; Tan and Greenwood, 2021; Zhu et al., 2021). These findings suggest that multilingual EMI environments involve more than English-medium teaching in non-Anglophone settings. Academic participation, institutional access, and social belonging are shaped by shifting communicative and cultural expectations that intensify uncertainty and psychological strain.

Language functions not merely as a medium of communication but as a structuring mechanism through which academic participation, institutional access, and psychological vulnerability are differentially negotiated within multilingual EMI environments. Prior research identifies difficulties with accent comprehension and communication anxiety in EMI (Hor and Jusoh, 2021; Lan et al., 2023; McDonough et al., 2022; Rathakrishnan et al., 2021), but the present findings explain why formal proficiency is a weak predictor of early stress in multilingual settings. Participants who met entry requirements still experienced high cognitive load when processing diverse English accents shaped by different linguistic backgrounds. This burden constrained participation, weakened supervisory interaction, and contributed to delayed progress. These patterns align with research on accent-based marginalization (Dovchin, 2020; Lam and Hale, 2024; Philips, 2021; Wang et al., 2019; Yeo et al., 2019) but are intensified in Malaysia, where linguistic diversity is routine rather than exceptional. In addition, students must navigate Malay-dominant administrative practices, creating continuous shifts between languages. Support is limited to generic academic English, which remains insufficient. Effective responses must ensure intelligibility across English varieties while reducing the cognitive burden of multilingual institutional navigation.

Supervision and pedagogy intensify stress within this multilingual and multicultural context. Delayed supervisory feedback, unclear milestones, and unstructured groupwork increase uncertainty and compound workload pressure (Han et al., 2024; Lian and Wallace, 2018; Lin et al., 2019; Tan and Greenwood, 2021; Wang, 2022; Zhu et al., 2021). These issues are not only procedural but also cultural. Norms of autonomy, indirect communication, and limited supervisory intervention may be interpreted differently by students from other educational traditions. When combined with linguistic complexity, these norms reduce clarity and predictability. From a self-efficacy perspective, ambiguous feedback weakens mastery experiences, exclusionary collaboration limits vicarious learning, and inconsistent expectations weaken social persuasion, leading to reduced efficacy and avoidance-oriented coping (Bandura, 1997; Kristensen et al., 2023; Sabouripour et al., 2021). Greater transparency in supervisory practices and clearer communication across linguistic and cultural boundaries can increase perceived control and support more adaptive coping (Rathakrishnan et al., 2022; Rosli and Saleh, 2023; Teh et al., 2023).

Sociocultural conditions further shape stress through the intersection of diversity and unequal access. Malaysia’s multicultural environment creates opportunities for interaction but does not guarantee inclusion. Experiences of discrimination and exclusion align with prior findings on psychological distress (Guo and Guo, 2021; Lalwani et al., 2022; Suh et al., 2019), yet here they are closely tied to linguistic positioning and group boundaries. Access to safety systems, institutional services, and everyday participation often depends on familiarity with local languages and cultural practices. Students with limited Malay and partial access to locally used English varieties, therefore, face barriers to engagement and protection. In academic contexts, unstructured groupwork allows linguistic and cultural comfort zones to shape collaboration, reinforcing segmentation. Under these conditions, multicultural coexistence does not necessarily translate into intercultural inclusion.

Participants’ coping practices reflected not only personal efforts to manage stress, but also adaptive responses to uneven academic and sociocultural conditions within Malaysia’s multilingual EMI context. Although participants adopted problem-focused, emotion-focused, and avoidance-oriented strategies consistent with previous research (Cao et al., 2018; De Moissac et al., 2020; Yu and Leung, 2023), these approaches frequently emerged in contexts characterized by institutional ambiguity, limited guidance, and uneven access to resources. Students relied heavily on self-management, co-national peer networks, digital communities, and emotional regulation because formal support systems were often experienced as unclear, linguistically demanding, or insufficiently responsive to their needs.

Importantly, these findings complicate simplified understandings of resilience in international education. Participants’ accounts of perseverance, self-regulation, and personal growth frequently coexisted with prolonged uncertainty, exhaustion, and psychological strain. In many cases, resilience appeared to develop not through strong institutional enablement, but through continued adaptation to conditions of limited clarity and uneven support. Overemphasizing resilience, therefore, risks normalizing unequal adaptation burdens by implicitly positioning international students as individually responsible for navigating structural barriers related to supervision, language mediation, academic opacity, and intercultural exclusion.

These findings suggest that improving psychological wellbeing in multilingual EMI environments requires more than strengthening students’ individual coping capacities. Greater institutional responsiveness is equally necessary. Clearer supervisory communication, more transparent progression structures, intelligibility-oriented language support, structured intercultural engagement, and more accessible multilingual administrative practices may reduce cognitive and emotional burden while strengthening students’ sense of predictability, participation, and belonging.

## 7 Conclusion

Psychological strain within multilingual EMI environments is closely associated with variations in clarity, intelligibility, and institutional access across linguistic and cultural boundaries. When academic expectations, communication practices, and institutional procedures remain insufficiently transparent across languages and cultural norms, uncertainty accumulates and gradually undermines students’ confidence, participation, and psychological wellbeing.

Interventions are therefore most effective when they reduce cognitive and procedural barriers embedded in everyday academic life. Greater transparency in supervision, clearer progression structures, intelligibility-oriented language support, and more inclusive approaches to groupwork and institutional communication can improve predictability and access. These changes strengthen students’ sense of control and enable more adaptive forms of coping.

Several limitations should be noted. The study draws on a small, context-specific sample and relies on cross-sectional self-reported data. Future research would benefit from longitudinal and comparative designs that trace how stress, self-efficacy, and adaptation develop over time and across different student groups. Incorporating perspectives from supervisors and institutions would further clarify how expectations and practices are negotiated in EMI settings. Mixed methods approaches may also strengthen explanatory power while retaining sensitivity to multilingual contexts.

Sustained improvement depends on addressing how institutional environments shape access, participation, and belonging. Evaluations of policy and practice should move beyond short-term stress reduction and consider whether changes enhance students’ capacity to engage, persist, and develop academically. Attention to these structural conditions is essential for improving student wellbeing and reconsidering how responsibility for adaptation is distributed within multilingual EMI higher education.

## Data Availability

The raw data supporting the conclusions of this article will be made available by the authors, without undue reservation.
